# Characterisation of the Selective Reduced Uteroplacental Perfusion (sRUPP) Model of Preeclampsia

**DOI:** 10.1038/s41598-019-45959-6

**Published:** 2019-07-02

**Authors:** J. S. Morton, J. Levasseur, E. Ganguly, A. Quon, R. Kirschenman, J. R. B. Dyck, G. M. Fraser, S. T. Davidge

**Affiliations:** 1 Faculty of Medicine and Dentistry, Dept. of Ob/Gyn, University of Alberta, Edmonton, AB T6G 2S2 Canada; 2 Faculty of Medicine and Dentistry, Dept. of Physiology, University of Alberta, Edmonton, AB T6G 2H7 Canada; 3 Women and Children’s Health Research Institute, Edmonton, AB T6G 2R3 Canada; 4 Faculty of Medicine and Dentistry, Dept. of Pediatrics, University of Alberta, Edmonton, AB T6G 2S2 Canada; 5 Division of Biomedical Sciences, Faculty of Medicine, Memorial University of Newfoundland, St. John’s, NL A1B 3V6 Canada

**Keywords:** Experimental models of disease, Translational research

## Abstract

Preeclampsia is a complication of pregnancy characterised by gestational hypertension, proteinuria and/or end organ disease. The reduced uteroplacental perfusion (RUPP) model, via partial occlusion of the lower abdominal aorta, mimics insufficient placental perfusion as a primary causal characteristic of preeclampsia. However, a major limitation of the RUPP model is that perfusion is reduced to the entire hindquarters of the rat resulting in hindlimb ischemia. We hypothesised that clipping the uterine and ovarian arteries in the selective (s)RUPP model would provoke signs of preeclampsia while avoiding systemic ischemia. Sham, RUPP or sRUPP procedures were performed in pregnant Sprague Dawley rats on gestational day (GD)14. On GD21 uterine blood flow was significantly reduced in both the RUPP and sRUPP models while aortic flow was reduced only in RUPP. Both models resulted in increased MAP, increased vascular oxidative stress (superoxide generation), increased pro-inflammatory (RANTES) and reduced pro-angiogenic (endoglin) mediators. Vascular compliance and constriction were unaltered in either RUPP or sRUPP groups. In summary, refinements to the RUPP model simultaneously maintain the characteristic phenotype of preeclampsia and avoid peripheral ischemia; providing a useful tool which may be used to increase our knowledge and bring us closer to a solution for women affected by preeclampsia.

## Introduction

Preeclampsia is defined as gestational hypertension along with one or more of the following signs: proteinuria, adverse conditions or severe complications affecting the CNS, cardiorespiratory, haematological, renal, hepatic and/or feto-placental systems^1^. Interestingly, preeclampsia is an almost uniquely human condition with very few cases observed in other species; those which have occurred have been primarily in higher primates^2–5^. This characteristic of preeclampsia poses a difficulty when it comes to investigating the mechanisms involved in the development of this pregnancy complication.

Through the years, several animal models have been developed which mimic signs of preeclampsia. While some models target single downstream mechanistic pathways such as endothelial nitric oxide synthase; soluble fms-like tyrosine kinase (sFlt); angiotensin auto-antibodies, or TNF-α; or use toxins, such as endotoxin, to induce hypertension; the reduced uteroplacental perfusion (RUPP) model is directed towards targeting a primary causal characteristic of preeclampsia, namely insufficient placental perfusion; reviewed in^6,7^. The RUPP procedure originated in the 1970’s when clipping of the uterine arteries and transection (complete ligation) of the ovarian arteries was performed in female baboons prior to mating; a method which was shown to induce hypertension and proteinuria during pregnancy^8,9^. This method was also applied to guinea pigs with similar results^10^. Experimental procedures were concurrently being tested to mimic compression of the abdominal aorta, which occurs in pregnant women lying in a supine position. Other studies were performed in various species such as rabbit, dog, and monkey, in which the aorta was experimentally constricted. Interestingly, aortic constriction itself mimicked signs of preeclampsia (toxemia) in humans, including hypertension and proteinuria^11–14^. While aortic compression *per se* is not directly relevant to the pathology of preeclampsia, the resultant restriction of blood flow to the uteroplacental units reflects the reduced blood supply that would be observed following insufficient spiral artery remodeling – a pathology that is considered to be a causal factor of preeclampsia; reviewed in^15^.

One of the first rat models of preeclampsia to be used was the spontaneously hypertensive rat (SHR), along with stroke-prone (SP-SHR) and heart failure (SHHF) strains, which develop increased blood pressure during pregnancy^16–18^. However, since the SHRs also develop hypertension prior to and independent of pregnancy, this complicates the model since preeclampsia is defined as the *de novo* onset of hypertension in pregnancy. The development of a rat model of preeclampsia, however, has many advantages over more expensive primate models. Therefore, by the early 1990’s reduced uteroplacental perfusion in rats began to be investigated as a potential model for the study of preeclampsia that was not specific to one mechanistic pathway^19,20^. These studies initially mimicked the aortic compression technique discovered in primates^21^. The model was then further developed by the Granger lab into the now well-known RUPP model with reduction of blood flow in both the abdominal aorta and uterine arteries^22–25^. This procedure has been well characterized and shown to produce many similarities to preeclampsia in humans; including hypertension, kidney glomerular morphology alterations, and intrauterine growth restriction, as previously detailed^26–30^.

Due to restriction of the abdominal aorta in this model, a common complication of the RUPP procedure is hindlimb ischemia which can progress to complete paraplegia and exclusion of test animals from the study (~8% of RUPP surgeries). This outcome is indicative of the fact that aortic compression, by design, occludes blood flow not only to the uteroplacental units but also to the entire hindquarters of the animal. This also raises concerns that the preeclamptic signs observed in this model, such as hypertension, are not specific to insufficient uteroplacental perfusion but may be caused by toxemia induced by systemically hypoxic tissues. This issue has been previously investigated by Schenone *et al*. who performed a brief study investigating the development of an improved model of preeclampsia, termed the selective RUPP model^31^. In their study, refining the RUPP model to restrict blood flow only to the uteroplacental units and not to the hindquarters increased mean arterial blood pressure, measured via tail cuff plethysmography, while they observed no effect on proteinuria, fetal or placental weight. Further, they demonstrated increased placental oxidative stress but not placental levels of sFlt. A limitation of the study by Schenone *et al*., however, was the lack of a RUPP group comparison and any assessment of blood flow effects, vascular function or maternal cytokine or oxidative stress analyses. To further characterize the sRUPP model and expand on this work, we hypothesised that restriction of blood flow specifically to the uteroplacental units – via clipping of the uterine and ovarian arteries – would induce the signs of preeclampsia in this model while avoiding potentially confounding effects caused by systemic ischemia.

## Methods

### Ethical approval

All protocols were approved by the University of Alberta Health Sciences Animal Policy and Welfare Committee and the University of Memorial Institutional Animal Care Committee (for a cohort of animals used in intraoperative blood flow measurements) in accordance with the guidelines of the Canadian Council on Animal Care and the Guide for the Care and Use of Laboratory Animals published by the US National Institutes of Health.

### Surgical models of preeclampsia

**Figure 1:**
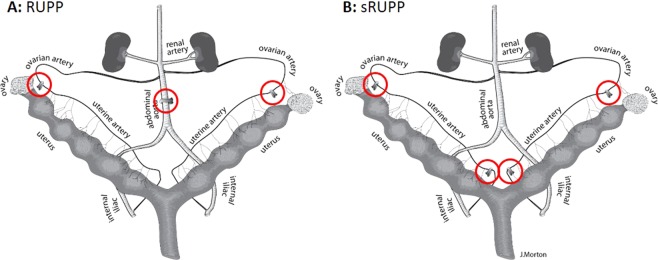
Schematic representation of the rat uterus showing placement of sliver clips in the (**A**) RUPP and (**B**) sRUPP procedures. In the RUPP model (**A**), silver clips of approximately 100 µm internal planar gap were placed on the ovarian arteries and a silver clip of approximately 230 µm internal planar gap was placed on the abdominal aorta. In the sRUPP model (**B**), silver clips of approximately 100 µm internal planar gap were placed on the ovarian and uterine arteries. During Sham procedures, silver clips were placed on abdominal fat and comparative manipulations were made of the arteries.

Three month old female Sprague-Dawley rats (Charles River, Wilmington, MA) were maintained on *ad libitum* standard rodent chow and filtered water in a 10:14 hour light:dark cycle. Following in-house acclimatisation, females were mated overnight and the presence of sperm in a vaginal smear the following morning was designated as gestational day (GD)0 of pregnancy. On GD14, rats were anaesthetized by inhaled isoflurane (3–4% induction, 1–3% maintenance; Pharmaceutical Partners of Canada, Ontario) and the abdominal cavity opened by a midline incision. For the RUPP procedure, a silver clip (ID 0.230 mm) was placed around the abdominal aorta above the iliac bifurcation and below the renal artery (Fig. 1A). To prevent compensatory flow via the ovarian arteries, silver clips (ID 0.100 mm) were placed around the left and right ovarian arteries between the ovary and the uterine horn. For the sRUPP procedure, the aortic clip was omitted and replaced with silver clips (ID 0.100 mm) around the left and right uterine arteries below the supply to the first fetus (Fig. 1B). Rats assigned to the Sham control group underwent comparative manipulations and placement of silver clips on intra-abdominal fat. Surgeries which resulted in maternal paraplegia or complete reabsorption of the fetuses were excluded from data analyses. All surgeries were carried out aseptically and buprenorphine (0.01–0.02 mg/kg) analgesia was administered for 48 hours following surgery. Two cohorts of animals were used; the first cohort underwent blood pressure, metabolic cage and *ex vivo* vascular function procedures while the second cohort underwent intraoperative blood flow analysis and additional *ex vivo* vascular function procedures. Tissues and offspring biometrics were collected from all groups.

### Blood pressure and proteinuria assessment

On GD5-7, the first cohort of animals (n = 7–8/group) underwent surgical insertion of a telemetric blood pressure recording device (TA11PA-C10; Data Sciences International, St. Paul, MN) under inhaled isoflurane anesthesia (3–4% induction, 1–2% maintenance). The TA11PA-C10 was implanted in the neck and a catheter was placed into the left carotid artery, advanced into the aortic arch, and secured. The body of the device was placed in a subcutaneous pocket at base of the neck. All surgeries were carried out aseptically and meloxicam (IM; 2–5 mg/kg) analgesia was administered for 48 hours following surgery. On GD11 animals were placed on DSI PhysioTel receivers and baseline physiological measurements obtained 10 min every hour for 10 consecutive days. On GD20, instrumented animals were placed in metabolic cages for the collection of 24-hour urine samples; blood pressure continued to be recorded during this process. Proteinuria was assessed in 24-hour urine samples using an albumin to creatinine ratio (ACR) assay kit (BioVision Inc., US) following the manufacturer instructions.

### Acute blood flow assessment

In the second cohort of animals, n = 5/group, blood flow in the abdominal aorta and uterine arteries was assessed on GD14 both pre- and post-clip placement. A midline incision was performed and the uterine horns exteriorised. A short, approximately 2–4 mm, section of the left and right uterine artery was cleared of all surrounding tissue and a flow probe (0.5PSL; Transonic, US) was placed on the exposed section and surrounded by ultrasonic gel (National Therapy Products Inc. Canada). Similarly, the abdominal aorta was cleared of surrounding tissue, as per the RUPP procedure, and a flow probe (1PRB or 1.5PRB; Transonic, US) was placed on the vessel and surrounded with gel. Flow rates were recorded uninterrupted for 10 minutes following which the RUPP, sRUPP, or Sham procedure was performed as previously detailed. Once the clips were in place, flow rates were recorded for another 5 minutes uninterrupted. After recording, flow probes were removed, and incisions closed using vicryl (4.0, muscle layer) and silk (4.0, skin layer) sutures.

### Vascular function

On GD21, rats were euthanized by exsanguination via excision of the superior vena cava under inhaled isoflurane anaesthesia. Mesenteric arteries were isolated for experimental procedures in ice-cold physiological saline solution (PSS), composition (in mmol/l): 10 HEPES, 5.5 glucose, 1.56 CaCl_2_, 4.7 KCl, 142 NaCl, 1.17 MgSO_4_, 1.18 KH_2_PO_4_, pH 7.4. Arteries were cleaned of all surrounding adipose and connective tissues and mounted on glass cannulae in a pressure myograph system (Living Systems, Burlington, VT) to allow isobaric measurements. Vessels were exposed to stepwise increases in pressure from 60 to 80 mmHg with regular changes of the PSS bathing media. Following equilibration, vasoconstrictor responses to phenylephrine (Phe: 0.01 to 100 µmol/l) or U46619 (U19: 0.001 to 10 µmol/l) were assessed. After a washout period, vasodilator responses to methylcholine (MCh: 0.001 to 100 µmol/l) were assessed in the absence or presence of the nitric oxide synthase inhibitor (*N*-nitro-L-arginine methyl ester hydrochloride [L-NAME]: 100 µmol/l).

Passive characteristics were assessed in the absence or presence of papaverine (0.1 µmol/l) and Ca^2+^-free PSS, using pressures from 0 to 140 mmHg. Circumferential wall stress and strain were calculated as previously described^32^. Arterial stiffness was determined using a tangential or incremental elastic modulus (E_inc_) obtained by determining the slope of the stress-strain curve where E_inc_ is directly proportional to k, the rate constant of an exponential curve was fitted to data from each animal. Any increase in the rate constant implies an increase in E_inc_ and, therefore, an increase in arterial stiffness while a decrease in the rate constant implies an increase in arterial elasticity.

### Placental culture

At euthanasia, the fetuses from each dam were sexed using anogenital distance, placentas from 3 female and 3 male fetuses were isolated, rinsed and placed into culture medium composition (44% DMEM, 44% F12, 1% Gentamicin, 0.4% penicilllin-streptomycin, 0.6% L-Glutamine, 10% FBS) in a 12-well plate. Placentas were cultured at 37 °C, 8% O_2_, and the culture media collected and replaced at 4, 24 and 48 hours. Levels of sFlt in the culture media was assessed using the Rat FLT1/VEGFR1 ELISA Kit, cat# LS-F21607, LifeSpan BioSciences, Inc.) following the manufacturer’s instructions.

### Cytokine analyses

A panel of cytokines was assessed in plasma samples using the rat Milliplex® MAP immunoassay (EMD Millipore) following the manufacturer’s instructions. The intra- and inter-assay coefficients of variation were ≤10% and 19.8% respectively. Cytokines were measured by Luminex® technology on a Bio-plex 200 system (Biorad). Five parameter logistic curve was used to calculate concentrations (pg/mL).

### Molecular analyses

Sections of thoracic aortae and mesenteric artery were embedded in optimal cutting medium (OCT) and snap-frozen in liquid nitrogen for subsequent analysis. Sections were cut at 8 μm, mounted on glass slides at −20 °C, and stored at −80 °C until use.

Slides were fixed in cold acetone for 10 mins and treated with 1 mg/ml sodium borohydride for 10 mins at room temperature to reduce auto-fluorescence. Following addition of blocking reagent (2% goat serum, 1% BSA, 0.1% Triton X-100), sections were incubated overnight at 4 °C in a humid chamber with primary antibody followed by 1 hour at room temperature with secondary antibody. The primary antibodies used were: nitrotyrosine, 1:100 rabbit polyclonal anti-nitrotyrosine, Milipore; collagen III, 1:50 mouse monoclonal COLIII, Santa Cruz; collagen I, 1:250 rabbit polyclonal COLI, Abcam; or elastin, 1:100 rabbit polyclonal ELN, Abcam. Secondary antibodies included: 1:250 AlexaFluor488 goat anti-mouse IgG (H + L), ThermoFisher; and 1:250 Chromeo546 goat anti-rabbit IgG (H + L), Abcam.

Subsequently, slides were washed to remove excess dye on the surface of the section, cover slipped with Vectashield mounting medium with DAPI, Vector laboratories, A subset of slides was thawed, washed thrice with Hank’s balanced salt solution (HBSS, containing calcium and magnesium), and incubated with 200 µM dihydroethidium (DHE) in HBSS for 30 mins at 37 °C.

One to four non-overlapping image fields were imaged using an IX81 Olympus fluorescence microscope. Images were processed using CellSens Dimensions 1.9 software and analysed using ImageJ. Mean fluorescence intensity was recorded and expressed as percent increase over control.

Expression levels of catalase, SOD1 and SOD2 were assessed by Western blot using the following primary antibodies: 1:50 mouse monoclonal eNOS, Biosciences; 1:50 rabbit polyclonal phospho-eNOS (Ser 1177), Cell Signalling; 1:4000 rabbit polyclonal β-actin, Abcam; 1:500 mouse monoclonal catalase, Santa Cruz; 1:1000 mouse monoclonal SOD1, Santa Cruz; 1:10000 mouse monoclonal SOD2, Santa Cruz or 1:2000 rabbit polyclonal β-actin, Abcam. Secondary antibodies included: 1:10000 IRDye 800CW donkey anti-rabbit IgG, IRDye 680RD donkey anti-mouse IgG and IRDye 680RD goat anti-rabbit IgG (Li-Cor Biosciences).

Briefly, thoracic aorta homogenates were prepared from 50–100 mg snap frozen samples. Tissue samples were lysed using buffer composition: 20 mM sodium fluoride, 1% NP-40 with 2 mM sodium orthovanadate, 1x protease inhibitor and 20 µg/ml phenylmethylsulfonyl fluoride (PMSF). Total protein was determined by the bicinchoninic acid assay and 150 µg of total protein was resolved on 12% SDS-polyacrylamide gels and transferred onto a nitrocellulose membrane. Protein bands were imaged, densitometry analysis performed using the Li-Cor Odyssey Imaging Systems v3.0 and were normalised to β-actin expression.

### Statistical analysis

Depending on normality of the data, parametric or non-parametric data were presented as mean ± s.e. or median (range), respectively, and were analyzed using GraphPad Prism software version 6.0. Mean arterial pressure and blood flow data were analysed using a two-way ANOVA with Sidak’s post-hoc test for multiple comparisons. For comparison of vascular function data, concentration-response curves were fitted to the Hill equation from which a sigmoid plot was generated by non-linear least squares regression analysis. Comparison of maternal body weight, proteinuria, litter size, cytokine, placental sFlt, vascular oxidative stress marker and vascular summary area under curve (AUC) data was made using a one-way ANOVA with Tukey’s (parametric data) or Kuskal-Wallis (non-parametric data) post-hoc test for multiple comparisons. A p < 0.05 was considered statistically significant.

## Results

### Pregnancy outcomes

**Figure 2:**
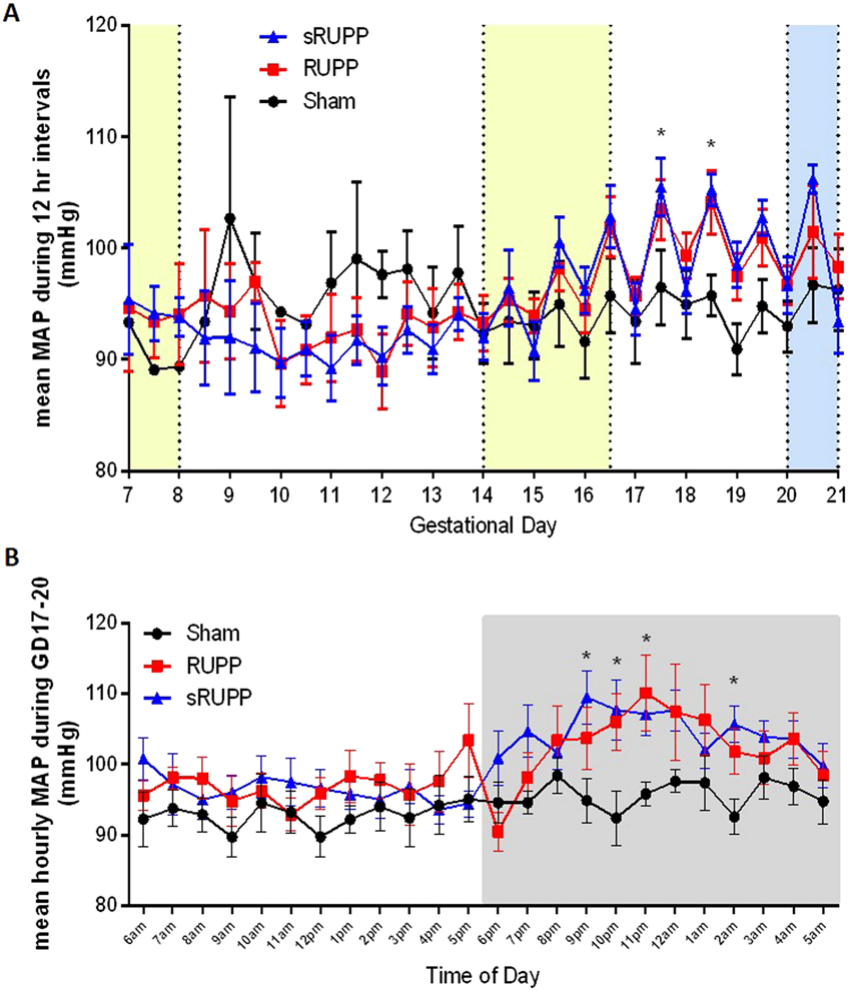
Mean arterial blood pressure recordings following instrumentation with telemetric TA11PA-C10 probes on GD5-7. (**A**) Data averaged over 12-hour intervals from GD7-21. Yellow bands indicate periods of analgesia medication. Blue band indicates period spent in a metabolic cage for urine collection. MAP was significantly elevated on GD17.5 and 18.5 following surgery on GD14. (**B**) Data for GD17-20 averaged over 1-hour intervals and plotted to display 24-hour cycles. In the Sham group MAP remained at around the level seen during daylight/inactive hours throughout the 24-hour period. In both RUPP and sRUPP groups, MAP was generally higher compared to SHAMs and found to be significantly different during night/active hours. Data analysed by two-way ANOVA with Sidak’s post hoc test; *p < 0.05 vs. Sham group.

Baseline mean arterial pressure (MAP) was unaltered between Sham, RUPP and sRUPP groups prior to surgery. Administration of buprenorphine analgesia can interfere with blood pressure analysis during the period in which it is given, therefore, GD14-16.5 was excluded from analyses. In addition, placement of rats into a metabolic cage may increase levels of stress and impact blood pressure hence data from GD20-21 was similarly excluded. Both RUPP and sRUPP groups demonstrated increased MAP on GD17.5 and 18.5, following recovery from surgery and administration of analgesia (Fig. 2A). Interestingly, both RUPP and sRUPP showed a greater diurnal variation in blood pressure than Sham animals. Indeed, Sham animals demonstrated no increase in MAP during the nocturnal hours of GD17-20 while both sRUPP and RUPP animals had similarly increased MAP during this period (Fig. 2B).

Both Sham and RUPP groups demonstrated similar levels of protein in 24-hour urine samples (ACR [mg albumin/g creatinine] Sham: 254 ± 26, RUPP: 238 ± 33). The modified sRUPP group had a mean ACR of 332 ± 37 mg albumin/g creatinine, however, this was not significantly increased compared to the Sham and RUPP groups.

Litter size was similarly reduced in the RUPP and sRUPP groups compared to Sham litters (Table 1). Correspondingly, maternal body weight was significantly reduced in the RUPP and sRUPP groups on GD21 (p < 0.01) while baseline body weights were not altered between the groups on GD0 or GD14. Placental and fetal weights were not different between the groups on GD21, however, an increase in the fetal/placental weight ratio – a measure of placental efficiency – was found in both RUPP and sRUPP groups.

**Table 1:** Maternal and fetal biometrics measured in early, mid and late gestation. Maternal body weight and litter size was decreased in both RUPP and sRUPP groups on GD21.

		Sham, n = 11	RUPP, n = 10	sRUPP, n = 14	One-way ANOVA or Kruskal-Wallis
Maternal body weight (g)	GD0	308 ± 7	315 ± 9	304 ± 8	
	GD14	354 ± 11	370 ± 10	373 ± 7	
	GD21	435 ± 7	**392** ± **12**^**††**^	**407** ± **9**^**†**^	**
Litter size (n)	GD14	15 (11–18)	16 (9–18)	16 (12–19)	
	GD21	14 (10–19)	**5 (1–13)** ^**†††**^	**6 (1–11)** ^**†††**^	***
Placental weight (g)		0.56 ± 0.02	0.48 ± 0.03	0.52 ± 0.01	
Fetal weight (g)		5.63 ± 0.22	5.32 ± 0.23	5.96 ± 0.10^€^	0.0529
Fetal/placental weight ratio (g/g)		10.2 ± 0.5	11.7 ± 0.5	11.6 ± 0.3	*
Crown-rump length/ab. girth ratio (mm/mm)		1.07 ± 0.03	1.09 ± 0.04	1.06 ± 0.04	

Data are presented as mean ± s.e. or median (range) and analysed by one-way ANOVA or Kruskal-Wallis test as appropriate for parametric or non-parametric outcomes. Bold and ^†^significant difference from Sham group. ^€^Significant difference from RUPP group.

### Blood flow in the aorta and uterine arteries

**Figure 3:**
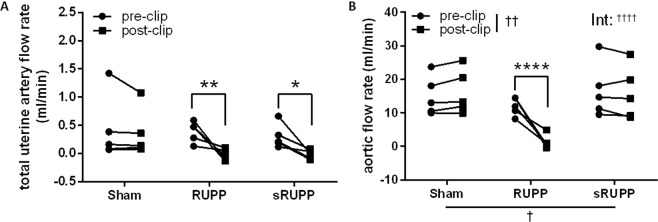
Blood flow rate in the (**A**) aorta and (**B**) total uterine blood flow as the sum of left and right uterine artery flow, at baseline (pre-clip) and following the Sham, RUPP or sRUPP procedure (post-clip). Total uterine blood flow was decreased by both the RUPP and sRUPP procedures while aortic flow was only decreased following the RUPP procedure. Blood flow in the sham operated group was unaltered in either vessel type. Data analysed by two-way ANOVA with Sidak’s post hoc test; *p < 0.05, **p < 0.01, ****p < 0.0001 vs. pre-clip values; ^†^p < 0.05, ^††^p < 0.01 group effect of experimental group or measurement period; ^††††^p < 0.0001 interaction effect between experimental group and measurement period.

Prior to any surgical intervention, blood flow in the aorta (Sham 15.1 ± 2.6, RUPP 11.9 ± 1.2, sRUPP 16.7 ± 3.6 ml/min) and total blood flow in the uterine arteries [right plus left] (Sham 0.42 ± 0.26, RUPP 0.39 ± 0.08, sRUPP 0.30 ± 0.10 ml/min) were similar between the groups. Following placement of clips on abdominal fat in Sham animals, blood flow was unaltered in the aorta (16.2 ± 2.9 ml/min) and uterine arteries (0.58 ± 0.50 ml/min). In animals receiving the RUPP procedure, blood flow was significantly reduced in both the aorta (1.4 ± 0.9 ml/min, p < 0.001) and uterine arteries (−0.03 ± 0.05 ml/min; p < 0.01). Clipping of uterine and ovarian arteries in the sRUPP procedure reduced blood flow in uterine arteries (−0.02 ± 0.04 ml/min, p < 0.05) but not the aorta (15.9 ± 3.5 ml/min), as expected (Fig. 3).

### Inflammatory cytokine production

Maternal plasma was assessed for an array of physiologically important inflammatory markers (Table 2). RANTES, a pro-inflammatory chemokine that is produced by macrophages and recruits leukocytes to sites of inflammation, was significantly elevated in both RUPP and sRUPP groups. In addition, the angiogenic membrane glycoprotein endoglin was significantly reduced in the RUPP groups. The pro-inflammatory mediator IL-1β, demonstrated a slight increase in only the sRUPP group that did not reach significance (p = 0.09.) No other pro- or anti-inflammatory cytokines among those tested were shown to be significantly altered.

**Table 2:** Cytokine levels in plasma from Sham, RUPP and sRUPP groups on GD21. Data are presented as mean (range) and analysed by the Kruskal-Wallis test for non-parametric data.

	Sham, n = 5	RUPP, n = 5	sRUPP, n = 5	Kruskal-Wallis test
***Pro-inflammatory***				
IFN-γ (pg/ml)	582 (367–1121)	481 (59–808)	659 (240–1260)	
IL-1α (pg/ml)	119 (63–208)	180 (116–197)	136 (102–318)	
IL-1β (pg/ml)	28 (9–41)	25 (12–49)	38 (11–68)	p = 0.0931
IL-17 (pg/ml)	78 (49–91)	85 (70–93)	83 (53–130)	
TNF-α (pg/ml)	9 (6–11)	8 (6–10)	6 (6–11)	
RANTES (pg/ml)	246 (73–320)	513 (318–1427)	657 (255–1703)	*
MCP-1 (pg/ml)	379 (213–515)	382 (131–413)	291 (253–574)	
***Pro- and anti-inflammatory***				
IL-6 (pg/ml)	212 (77–629)	224 (154–309)	350 (131–466)	
***Anti-inflammatory***				
IL-10 (pg/ml)	104 (36–203)	84 (10–143)	56 (2–143)	
***Angiogenesis***				
VEGF (pg/ml)	106 (60–126)	123 (79–182)	85 (56–174)	
sFlt (ng/ml)	1.26 (0.76–2.65)	0.93 (0.56–2.14)	0.93 (0.65–2.00)	
PlGF (pg/ml)	0.29 (0.14–0.85)	0.10 (0.04–0.98)	0.19 (0.09–0.75)	
Endoglin (ng/ml)	0.84 (0.65–1.21)	0.59 (0.35–1.10)	0.69 (0.40–0.76)	*

### Vascular function

On GD21, passive characteristics of the mesenteric arteries, determined by the cytoskeletal components of the vascular wall, were unaltered between the groups (circumferential stress/strain rate constant, k values: Sham 6.9 ± 1.0; RUPP 8.7 ± 1.2; sRUPP 6.1 ± 0.7). Expression of the cytoskeletal components themselves were also unaltered between the groups (Collagen I: Sham 1.53 [1.02–2.29]; RUPP 2.62 [1.71–4.33]; sRUPP 2.25 [1.43–3.41] MFI a.u., Elastin: Sham 2.48 [1.43–2.97]; RUPP 2.29 [1.20–3.18]; sRUPP 2.24 [1.08–2.64] MFI a.u.).

**Figure 4:**
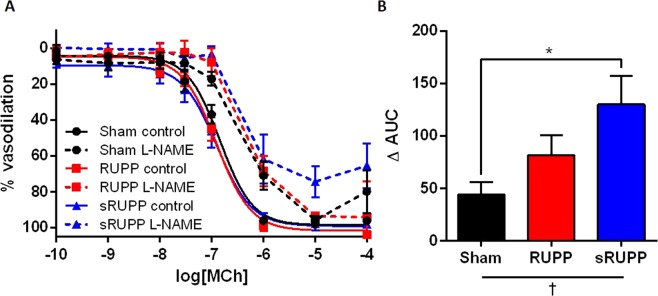
(**A**) Mesenteric artery vasodilator responses to MCh in the absence or presence of the nitric oxide synthase inhibitor, L-NAME (10 µmol/l). (**B**) Summary delta (difference between curves with and without L-NAME) area under the curve (Δ AUC) data for each group. The contribution of nitric oxide to vasodilation was significantly increased in the sRUPP group. AUC data analysed by one-way ANOVA with Tukey’s post hoc test; *p < 0.05 vs. Sham group; ^†^p < 0.05 group effect of experimental group.

Mesenteric artery vasoconstrictor responses to Phe were unaltered by either RUPP or sRUPP surgery (pEC_50_: Sham 6.15 ± 0.06; RUPP 5.99 ± 0.04; sRUPP 6.09 ± 0.05). Responses to the thromboxane mimetic (U46619, pEC_50_: Sham 7.66 ± 0.19; RUPP 7.43 ± 0.08; sRUPP 7.45 ± 0.05) or active responses to increases in intraluminal pressure (0 to 140 mmHg; data not shown) were similarly unaltered. While overall vasodilator responses to MCh were similar between the groups, there was a significant increase in the contribution of nitric oxide to vasodilation in the sRUPP group (Fig. 4).

### Vascular oxidative stress

**Figure 5:**
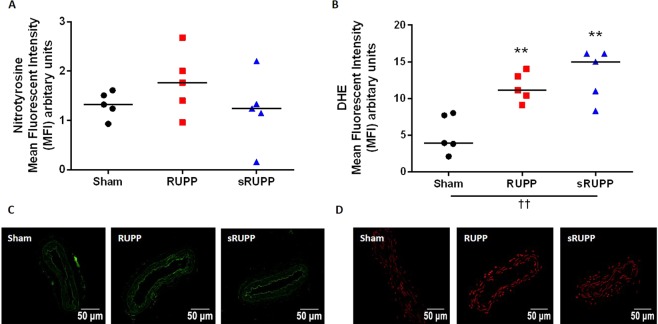
(**A**) Nitrotyrosine levels, a footprint of peroxynitrite production, in the mesenteric arteries of Sham, RUPP and sRUPP groups was unaltered. (**B**) Superoxide production, as measured by DHE staining, was increased in both RUPP and sRUPP groups. Representative images of nitrotyrosine (**C**) and DHE (**D**) stained sections of mesenteric artery. Data analysed by one-way ANOVA with Tukey’s post hoc test; **p < 0.01 vs. Sham group; ^††^p < 0.01 group effect of experimental group.

Levels of the antioxidants catalase and superoxide dismutase (SOD1 and SOD2) in the thoracic aorta were unaltered at GD21 of pregnancy (Table 3). While nitrotyrosine, a footprint of peroxynitrite generation, was unaltered between the groups, DHE, a measure of superoxide production, was significantly increased in aorta from both RUPP and sRUPP groups (Table 3). Similarly, in the mesenteric arteries nitrotyrosine was unaltered and DHE was significantly increased in both RUPP and sRUPP groups (Fig. 5).

**Table 3:** Analysis of oxidative stress and antioxidant levels in the thoracic aorta.

	Sham, n = 5	RUPP, n = 5	sRUPP, n = 5	Kruskal-Wallis test
***Antioxidants***				
Catalase (a.u.)	3.91 (1.97–4.91)	1.86 (1.59–3.58)	2.49 (0.88–3.47)	
SOD1 [Cu-Zn] (a.u.)	9.21 (5.69–13.32)	13.67 (7.56–22.70)	12.81 (9.48–18.63)	
SOD2 [Mn] (a.u.)	17.66 (9.44–27.74)	13.72 (9.00–18.24)	16.55 (6.25–17.23)	
***Oxidative stress***				
Nitrotyrosine (MFI; a.u.)	2.53 (1.16–3.48)	1.85 (1.05–3.36)	1.95 (1.12–3.63)	
DHE (MFI; a.u.)	3.9 (2.1–8.0)	**11.1** (**9.0**–**14.0)**^**††**^	**15.0** (**8.3–16.1)**^**††**^	**

Data are presented as median (range) and analysed by Kruskal-Wallis test as appropriate for non-parametric outcomes. Bold and ^††^significant difference (p < 0.01) from Sham group.

### Placental sFlt expression

**Figure 6:**
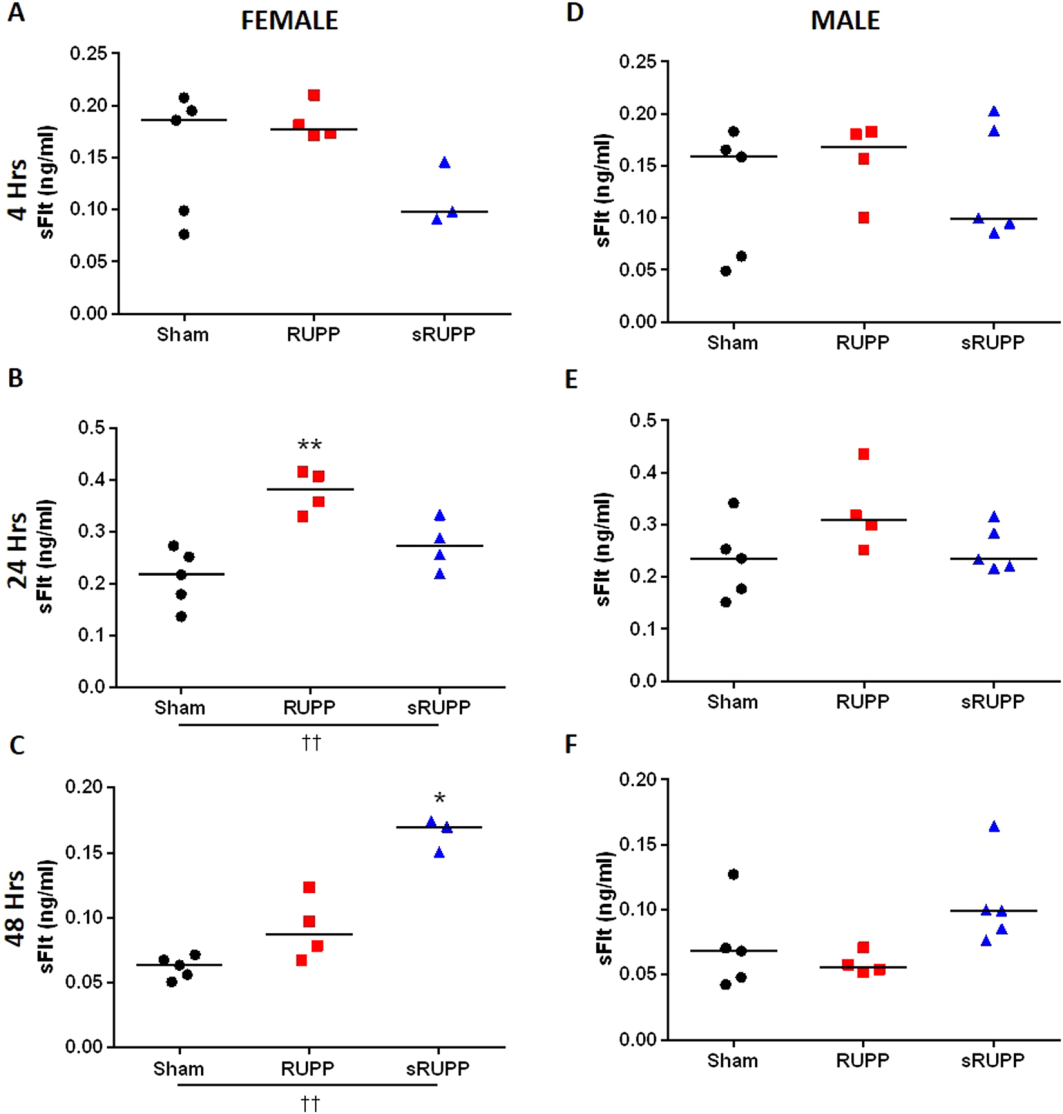
Placental production of sFlt measured in bathing media at 4, 24 and 48 hours of culture in placentas from female (**A–C**) and male (**D**–**F**) fetuses from each Sham, RUPP and sRUPP dams cultured on GD21 of pregnancy (individual data points show mean levels from each dam). sFlt levels were significantly increased in female RUPP placentas following 24 hours of culture and in female sRUPP placentas following 48 hours of culture. No changes in sFlt levels were observed in cultures of male placentas from any group. Data analysed by one-way ANOVA with Tukey’s post hoc test; *p < 0.05, **p < 0.01 vs. Sham group; ^††^p < 0.01 group effect of experimental group.

Release of sFlt from either male or female placentas into the culture medium was unaltered between groups at 4 hours of culture (Fig. 6). In media collected between 4–24 hours of culture, placental sFlt release was increased in female placentas from RUPP dams (Fig. 6B), but not in either male placentas or placentas from sRUPP dams. After an additional 24 hours of culture, placental release of sFlt was increased in female, but not male, placentas from sRUPP dams (Fig. 6C).

## Discussion

While the RUPP model has been successfully used to delineate many aspects of preeclampsia, it has a significant limitation. The traditional rat model involves the placement of restrictive clips on both ovarian arteries and on the abdominal aorta and, while this results in the desired outcome of reduced uteroplacental perfusion, it also causes a reduction of blood flow to the hindquarters of the rat resulting in hindlimb ischemia. The presence of an aortic clip also complicates the evaluation of potential treatment strategies since therapeutics targeted towards treating outcomes of placental ischemia will also influence the systemic ischemia effects of the model. In addition, the presence of an abdominal aortic restriction prevents the study of long-term postpartum conditions following preeclampsia due to continued growth of the animal which will, consequentially, increase the impact of the aortic clip on peripheral blood flow. Given these limitations, the current study aimed to further characterise a refined RUPP model, the sRUPP model initially proposed by Schenone *et al*., which does not include restriction of aortic blood flow. Our study has demonstrated preservation of the signs of preeclampsia in a model where aortic restriction was eliminated and replaced with restriction at both ends of the uterine vasculature supplying blood to the uteroplacental units. The sRUPP model showed considerable consistency with the less selective RUPP version and was in line with the known signs of preeclampsia in patients. The sRUPP model is supported as a refinement which allows additional investigation of the condition of preeclampsia; including postpartum effects and assessment of potential therapeutic strategies.

The primary limitation of the RUPP model, namely peripheral ischemia, was assessed via measurement of blood flow in both the aorta - distal to the location of clip placement - and of the resultant blood flow to the uterine vasculature. As intended, clipping the abdominal aorta in the RUPP model reduced peripheral blood flow by approximately 84%. During the initial development of the RUPP model, Abitbol *et al*. used a flow probe and silk ligature to accurately reduce aortic flow by 35–40% but found this technique too labour intensive to continue^33^. Interestingly, this study concluded that using a clip size of approximately 0.431 mm would achieve the desired reduction in blood flow while in subsequent investigations using the model, the most consistently used clip size was 0.203 mm reviewed in^6^. In our own previous studies, we modified the clip size to 0.230 mm in order to more frequently preserve litter sizes of one or more pups. These reduced clip sizes would explain the greater reduction in blood flow which was observed acutely following clip placement. Due to the considerable restriction of aortic blood flow, the established RUPP model is also known to result in reduced blood flow to the heart, stomach, intestine and skeletal muscle (right hindlimb), and decreased cardiac output^34,35^. Acute decreases in aortic blood flow after clip placement may be tempered by some peripheral compensation both post-operatively and following recovery. Partial restoration of aortic flows following the RUPP procedure is supported by the more moderate decreases in muscle perfusion observed previously at GD20^34^. As expected, neither the Sham nor sRUPP procedures impacted aortic blood flow.

The intent of both the RUPP and sRUPP models was to reduce perfusion of the uteroplacental units to mimic insufficient trophoblast invasion in the placenta, a feature which is central to the pathophysiology of preeclampsia. Correspondingly, total uterine blood flow was dramatically reduced in both the RUPP and sRUPP models. Interestingly, the resultant mean uterine flow in both RUPP and sRUPP groups had a negative directionality relative to baseline flow. The reversal of flows observed following restriction of the ovarian and uterine (sRUPP) or aortic and uterine (RUPP) vessels indicate a shift in the predominant direction of flow through the uterine loop but are not indicative of a complete lack of blood flow to the uteroplacental units. The uterine vasculature consists of a loop which is created by branching from the abdominal aorta to the common iliac, then to the anterior division of the internal iliac artery, and subsequent branching to the uterine arteries (Fig. 1). At the distal end of the uterus, the uterine artery combines with the ovarian artery which has its origin in the abdominal aorta below the level of the renal artery. The supply of blood flow to individual uteroplacental units, therefore, can originate primarily from either the uterine or ovarian ends of this loop and may be influenced by the number of pups in the uterine horn, the relative location of the flow probe and other factors such as venous drainage. The reductions in blood flow demonstrated in this study indicate that substituting the aortic clip with clips on the proximal uterine arteries in the sRUPP model was successful in reproducing the reduced uteroplacental perfusion outcome while avoiding any impact on peripheral tissue or organ perfusion.

An important aim of this study was to determine if more selective restriction of blood flow would maintain the phenotypic outcomes required to provide a useful model of preeclampsia. One of the primary defining characteristics of preeclampsia is hypertension, however, studies of MAP in the RUPP model have been restricted to few discreet measurements during pregnancy. For example, many studies assess MAP as a terminal outcome on GD18-20 and both ourselves^27,36,37^ and others^38–42^, reviewed in^6^ have consistently shown increased blood pressure in the RUPP model at this stage of gestation. In an uncomplicated rat pregnancy, blood pressure has been shown to decrease around GD18-19^43^ or GD11-15^44^. In the current study, we performed telemetric assessment of blood pressure from GD5 through to GD21. Certain periods of blood pressure recording were excluded from analysis due to the potential for data to be affected by analgesia administration^45^ or the stress of being placed in a novel environment such as a metabolic cage^46^. Despite these exclusions, the current study represents the most complete assessment of blood pressure available in the RUPP and sRUPP models to date. Interestingly, data in Sham animals showed a maintenance of blood pressure throughout pregnancy with only minor diurnal variations. Following surgery in both RUPP and sRUPP animals, diurnal variation in blood pressure was significantly increased during active hours in both of these groups. Blood pressure during non-active periods was higher, though not significantly, in RUPP and sRUPP groups compared to SHAM suggesting that observed diurnal differences may be caused by the overall increase in blood pressure in RUPP and sRUPP animals and not limited to the increases observed during active hours. These findings indicate that a normal adaptation of pregnancy to maintain blood pressure at a reduced level was lost following restriction of uteroplacental perfusion via either procedure.

Proteinuria is a variable outcome of the RUPP model in rats. Of the studies which have measured urinary protein, some have found increases^38,47^, some unchanged^22,40,48^, while others demonstrated a non-significant tendency to be increased^27,49^. In the current study, we also did not observe any significant increases in urinary protein in either the RUPP or sRUPP groups, although there appeared to be a tendency towards an increase in sRUPP animals. It could be speculated that the short duration of gestation in the rat reduces the likelihood of inducing severe enough renal changes in animals to consistently observe proteinuria in this species.

Heightened blood pressure in preeclampsia is thought to be initiated by a cascade of events beginning with decreased invasion of the trophoblast cells of the placenta leading to insufficient spiral artery remodelling and reduced perfusion compared to a normal pregnancy^50,51^. Measures of circulating pro-angiogenic factors, such as VEGF^48,52–57^, have previously been shown to be reduced in the RUPP model while circulating anti-angiogenic factors, such as sFlt^39,40,48,53,55,58^, were increased. Placental expression of sFlt was shown to be increased in human placental explant^48^ and rat placenta^54^ but, in another study, was unaltered in mouse placenta^40^. In the current study, plasma levels of VEGF, sFLT and PlGF were unaltered, however, the pro-angiogenic factor sEndoglin showed an overall tendency to be reduced in both RUPP and sRUPP groups. Production of sFlt by female, but not male, placentas was demonstrated to be increased in culture; placentas from RUPP dams showed an increased sFlt production after 24 hours in culture while placentas from sRUPP dams showed an increase after 48 hours of culture. Previous studies have also demonstrated increased levels of sFlt in women carrying female fetuses with either healthy pregnancies or those complicated by preeclampsia^59^, however, while no sex differences were observed in placental sFlt levels in healthy pregnancies in mice^60^, sFlt gene expression was shown to be increased in female mouse placentas^61^. The clear sex difference in sFlt production may lead to a greater impact on the development of female fetuses whose placentas have increased anti-angiogenic factors, however, it is also possible that female fetuses are more resistant to normally elevated sFlt levels while male fetuses are impacted to a greater extent by pathological levels. Indeed, pregnant mice treated with sFlt during pregnancy gave birth to male offspring with significantly elevated blood pressure while their female offspring were unaffected^62^. It is unknown what could cause the temporal differences between RUPP and sRUPP release of sFlt; however, in terms of a model of preeclampsia, both are in line with human placental explant data.

In addition, factors released from a stressed placenta are thought to trigger an oxidative stress response in the maternal vasculature. Increased systemic oxidative stress is a central hypothesis to the development of vascular dysfunction in preeclampsia, which primarily affects endothelial function. Many studies using the RUPP model have shown increased vascular^37,63,64^, maternal blood^48,53,63,65^, renal^66^, placental^48,49,52,53,66–68^, and amniotic fluid^69^ oxidative stress. Concurrently, the renal^55,68^, maternal blood^47,53^, placental^49,55^, and amniotic fluid^55^ antioxidant capacity has been shown to be reduced. This mirrors data from human patients with preeclampsia; reviewed in^7^. In the current study, increased aortic and mesenteric artery oxidative stress, through superoxide but not nitrotyrosine levels, was confirmed in both RUPP and sRUPP animals, however, we did not observe any changes in the expression of antioxidants in these vascular beds.

The circulation of factors released from a stressed placenta during preeclampsia initiate an inflammatory response in the maternal vasculature. For example, circulating TNF levels have been shown to be increased in women with preeclampsia; reviewed in^7^. This finding has been confirmed in previous studies in the RUPP model of preeclampsia^39,70^. In the current study, plasma levels of the pro-inflammatory cytokine RANTES was demonstrated to be increased in both RUPP and sRUPP groups while IL-1β showed a slight but non-significant increase in the sRUPP group. In line with these findings, Clayton *et al*. demonstrated increased brain IL-1β and RANTES levels in RUPP animals at 2 months postpartum along with increases in IL-4, IL-12, IL-17, IL-1α, leptin, MIP2 and a decrease in IL-8^71^. IL-6 has also been shown to be increased in maternal plasma following the RUPP procedure^39^.

Following the demonstration of increased MAP in RUPP and sRUPP animals, an assessment of structural, constrictor and dilator components of vascular function was made to determine if these may constitute mechanisms downstream of placental ischemia and maternal vascular oxidative stress and inflammation which lead to elevated blood pressure. The compliance of mesenteric arteries, a vascular bed that is important in the acute regulation of blood pressure, was assessed via changes in intraluminal pressure and neither passive stretch nor active myogenic responses to pressure changes were found to be altered. Correspondingly, expression levels of collagen and elastin, components of vascular structure which confer compliance and elasticity, were also found to be unaltered. In one previous study, collagen but not elastin levels were found to be increased in the uterus, placenta and aorta of rats which had undergone the RUPP procedure^72^. In the present study, although collagen expression tended to be higher in RUPP and sRUPP groups compared to Sham, no significant difference was found. Furthermore, the functional vascular compliance showed no evidence of increased vascular stiffness indicating that the increased blood pressures observed in the RUPP and sRUPP groups are not explained by changes to vessel structure.

Investigation of vasoconstrictor responses to Phe or U46619 showed no differences in the RUPP or sRUPP groups. Increased vasoconstriction of mesenteric arteries from RUPP animals has been previously demonstrated in our own studies in response to big ET-1^73^ but not Phe^27^ while other investigators have demonstrated increased mesenteric artery responsiveness to Phe^74^, KCl^74,75^, AngII^74^, ET-1^75^, or myogenic responses^76^. In another investigation, maximal mesenteric artery responses to Phe, ET-1 and AngII were unaltered but underlying Ca^2+^ sensitisation pathways were changed in RUPP animals in a manner which potentially protected these vessels against excessive vasoconstriction^77^. In uterine arteries from RUPP animals, increased responsiveness to Phe, U46619 and AngII^76^, or increased myogenic activity without changes in responses to Phe or U46619^78^ have been observed. Alterations in vasodilator pathways have also shown variable outcomes with some studies showing decreased responses to vasodilators^30,47,64,76^ while others showed no change^56,74^. In our previous studies we have observed changes in underlying vasodilator pathways such as the platelet-activating factor receptor^37^, or lectin-like oxidised LDL receptor^27^ without any major impact on vasodilator capacity to MCh. Similarly, in the current study responses to MCh were unaltered while there was an increase in the contribution of nitric oxide to vasodilation in the sRUPP group. The nitric oxide pathway was shown to be impacted in dilation of the thoracic aorta from RUPP animals in our previous work, however, in that vascular bed the nitric oxide contribution to vasodilation was reduced^27^. Therefore, as with many complex conditions such as preeclampsia, the outcomes can be heterogeneous and vascular bed-dependent and, thus, require greater numbers of studies in order to determine the likely pathological mechanisms of the condition.

In summary, while the pathophysiology of preeclampsia in both humans and the animal models used to investigate the condition is highly heterogeneous, both the RUPP and sRUPP models serve as representative animal models providing useful tools in which to study the mechanisms involved. The refinements proposed (sRUPP model), further improve this model of preeclampsia by simultaneously maintaining the characteristic phenotypes of the condition while avoiding a critical limitation of the established RUPP version, namely peripheral ischemia. In particular, the sRUPP variation may provide a model in which the maternal implications of a preeclamptic pregnancy can be investigated in the longer postpartum period. Women with a history of preeclampsia are known to have an increased risk of having a second pregnancy complicated by preeclampsia^79^ and, in addition, have been shown to have persisting endothelial dysfunction which may predispose them to later cardiovascular disease; reviewed in^80,81^; hence this is an important area of investigation. Further, the sRUPP model provides a useful tool for the investigation of therapeutics aimed at interrupting the feed-forward cycle in which insufficient trophoblast invasion causes a stressed placenta that releases factors into the maternal circulation initiating a vascular inflammatory and oxidative response further impacting the placenta as it is exposed to these effects. The sRUPP and RUPP models have an advantage in that they model the reduced placental perfusion considered as a central initiating factor in preeclampsia and, thereby, are not reliant on a single specific mechanistic pathway as in some other models of the condition. The refinement in the sRUPP model further removes any complicating effects of peripheral tissue or organ ischemia. The sRUPP model, therefore, provides a useful tool which may be used to further advance our knowledge of this pregnancy disorder and bring us closer to a solution for women affected by this condition.

## Data Availability

All data generated or analyzed during this study are included in the published article.
